# Effects of Ɛ-Polylysine Combined with Plant Extract on the Microbiological and Sensory Qualities of Grapes

**DOI:** 10.3390/foods14030516

**Published:** 2025-02-05

**Authors:** Qixin Feng, Chengzhi Zhu, Peng Zhou, Jinlong Yao, Yihong Bao, Zhijun Zhao

**Affiliations:** 1 College of Life Sciences, Northeast Forestry University, Harbin 150040, China; fengqixin0313@163.com (Q.F.); 2021115041@nefu.edu.cn (C.Z.); 2 Laboratory of Biorefinery, Shanghai Advanced Research Institute, Chinese Academy of Sciences, Shanghai 201210, China; 211310154@st.usst.edu.cn (P.Z.); 223392702@st.usst.edu.cn (J.Y.); 3 School of Health Science and Engineering, University of Shanghai for Science and Technology, Shanghai 200093, China

**Keywords:** grape, perilla extract, cinnamon extract, Ɛ-polylysine, microbial control, weight loss

## Abstract

Grapes are prone to rot and deterioration during storage, seriously affecting their food value. The effects of five extracts, cinnamon, perilla, green tea, pomegranate peel, and ginger, on the microbial growth, weight loss, and sensory quality of grapes were investigated using colony counting and sensory scoring methods. The results showed that perilla and cinnamon extracts had the best effect on maintaining the overall freshness of grapes on the 35th day of storage. The sensory scores were 82 and 80, respectively, and the number of microorganisms was below 6.13 log CFU/g. Further studies revealed that the combination of perilla and cinnamon extracts with Ɛ-polylysine resulted in better inhibition of microbial growth, reduced weight loss, maintained grape quality, and extended storage period to 40 days. An analysis of the active ingredients of the perilla and cinnamon extracts revealed that both extracts contained active antioxidant and antimicrobial ingredients, such as protocatechuic acid, coumaric acid, protocatechuic aldehyde, and rutin. The active ingredients of the perilla extract also included luteolin and apigenin, and those of the cinnamon extract included pinocembrin and epicatechin. These ingredients were deduced to have contributed to preserving the freshness of grapes by the plant extracts.

## 1. Introduction

Table grapes are thin-skinned and juicy, making them highly susceptible to spoilage. In addition, their shelf life is usually shortened by microbial contamination, hardness loss, berry abscission, stem discoloration, and other adverse factors [1]. Currently, common methods for controlling grape rot include SO_2_ sterilization [2] and ClO_2_ sterilization during low-temperature storage. However, SO_2_ and ClO_2_ residues can pose potential risks to human health. Therefore, efforts are being made to find new biological preservation methods to extend the storage period of grapes. A study on the preservation of grapes with chitosan coatings enriched with turmeric and green tea extracts revealed that a coating containing turmeric extract inhibited the value-added gray mold fungus of grapes, and a coating containing green tea extract extended the storage period of grapes from 4 to 8 days [3].

Plant extracts contain a variety of bioactive compounds, including flavonoids, phenols, terpenoids, alkaloids, and polysaccharides. These active ingredients have good antibacterial and antioxidant properties. The antimicrobial substances of plant extracts disrupt the cell membranes of microorganisms and can inhibit or reduce the activity of microorganisms on the surfaces of fruits and vegetables, thus acting as bacteriostatic or bactericidal agents [4]. The application of plant extracts to fruits and vegetables can increase the antioxidant activity of fruits and vegetables, inhibit the activity of enzymes such as polyphenol oxidase (PPO) and peroxidase (POD), maintain the reduced state of fruits and vegetables, and prolong the shelf life of fruits, vegetables, and other agricultural products [5]. For example, *Nigella sativa* L. extract was used to treat grapes, and the results showed that the control group had black spots on the surface on the 5th day of storage, while the experimental group had black spots on the surface on the 7th day of storage, which prolonged the storage period of grapes [6]. The perilla alcohol extract is rich in perillaldehyde, which inhibits Gram-negative and Gram-positive bacteria and fungi. Previous studies have reported that when ginger is treated with perillaldehyde, the minimum inhibitory concentration of perillaldehyde on *Fusarium graminearum* (*F. graminearum*) in ginger is 240 μL/L, which disrupts the energy supply and metabolism of the mycelium [7]. Cinnamon extract contains cinnamaldehyde, polysaccharides, diterpenes, polyphenols, and flavonoids, which have some antibacterial and antioxidant activities [8]. Catechins in green tea are the most common and abundant polyphenolic compounds with broad-spectrum antimicrobial activity against both Gram-positive and Gram-negative bacteria [9]. Pomegranate peel extract has active substances such as garnetin, ellagic acid, and gallic acid, which have significant antioxidant effects [10]. Ginger extract has active substances such as curcumin, paradols, shogaol, alkaloids, flavonoids, and zingerone, which have inhibitory effects on bacteria such as *Escherichia coli* (*E. coli*) [11].

Ɛ-Polylysine (Ɛ-PL) is a novel, natural, potent bacteriostatic and fungicidal substance produced by the fermentation of *Streptomyces albicans* through the amide bond formed between the α-carboxylic group of L-lysine and the Ɛ-amino group [12]. Currently, Ɛ-PL has been shown to have good bacteriostatic effects against Gram-positive bacteria, Gram-negative bacteria, molds, yeasts, and viruses [13]. There are many studies on the use of polylysine to preserve fruits and vegetables, but few studies on the use of polylysine in combination with plant extracts.

The present study investigated the effects of cinnamon (CE), perilla (PLE), green tea (GTE), pomegranate peel (PE), and ginger (GE) extracts on the microbiological, quality, and sensory qualities of grapes. Based on the significant antioxidant properties of the plant extracts and the bacteriostatic properties of Ɛ-polylysine, the effects of the perilla and cinnamon extracts combined with Ɛ-polylysine were further investigated. Finally, the preservation mechanism of the plant extracts was analyzed through the determination of the active ingredients of the perilla and cinnamon extracts.

## 2. Materials and Methods

### 2.1. Main Instruments and Equipment

Electronic balance (ME3002; Mettler-Toledo Instruments (Shanghai Co., Ltd., Suzhou, China); refrigerator (BCD-312WDPV; Shanghai Haier Group Co., Ltd., Shanghai, China); purification bench (SW-CJ-2FD; Suzhou Purification Equipment Co., Ltd., Suzhou, China); electrothermal incubator (ZXDP-A2160; Shanghai Zhicheng Analytical Instrument Manufacturing Co., Ltd., Shanghai, China); vertical pressure steam sterilizer (LDZX-75KBS; Shanghai Shenan Medical Instrument Factory, Shanghai, China); sterile homogenizer (SCIENTZ-11L; Ningbo Xinzhi Biotechnology Co., Ltd., Ningbo, China); Centrifuges (LC-LX-H185C 18,500 rpm Shanghai Lichen Co., Ltd., Shanghai, China); ultrasonic bath (KQ-300DE; Kunshan Ultrasonic Instrument Co., Ltd., Kunshan, China); UPLC (ACQUITY Premier; LC-MS; Waters Corporation).

### 2.2. Sample Pretreatment

Plant extracts were dissolved in distilled water to a final concentration of 0.60 g/L, Labeled as solution 1. Ɛ-PL was then dissolved in solution 1, leading to the final concentrations of Ɛ-PL, which were 0.20, 0.40, 0.60, 0.80, and 1.00 g/L. Luria–Bertani (LB) medium composition: 1.00% peptone, 0.50% yeast extract, 1.00% NaCl, pH 7.00.

Sunny rose grape berries of uniform cob size and maturity, without mechanical damage, were selected for treatment, and after picking, they were divided into experimental and control groups. The experimental group was immersed in different types of water-soluble plant extracts (purchased from Xuancheng Baica Pharmaceutical Co., Ltd. Lot No. T2210208) as well as the Ɛ-PL composite (purchased from Zhejiang Silver-Elephant Bio-engineering Co., Ltd.) for 10 min. The plant extract species were cinnamon (CE), perilla (PLE), green tea (GTE), pomegranate peel (PE), and ginger (GE) extracts and the mass concentration was set at 0.60 g/L. The control group was immersed in distilled water for 10 min. The samples were removed, drained, and stored in a refrigerator with a temperature of 4 °C and a relative humidity of 40%~60% in the dark. The total number of microorganisms and weight loss were determined every 5 days.

### 2.3. Effects of the Plant Extract Type on the Microbiological and Sensory Qualities of Grapes

#### 2.3.1. Effects of the Plant Extract Species on the Total Number of Colonies

Determination of the total number of microbial colonies according to the method of Zhu et al. [14]. A total of 10 g of grape sample that had been soaked in a solution of plant extracts at a concentration of 0.60 g/L was accurately weighed and poured into a homogenizing bag. After 90 mL of sterilized saline was added, the sample was tapped with a tapping homogenizer for 1 min. A total of 1 mL of homogenate was subjected to a 10-fold series of dilutions. The total number of colonies was counted on LB medium at 37 °C for 48 h before enumeration. All counts were performed in triplicate. The error between parallel samples was less than 7.70%. The results were expressed as logarithmic CFU/g.

#### 2.3.2. Effects of the Plant Extract Type on the Weight Loss of Grapes

Weight loss was tested as reported by Zhu et al. [14]. The effects of the plant extract species on the rate of weight loss of grapes were investigated. The weight loss rate of the grapes was calculated by dividing the initial mass minus the mass measured during storage by the initial mass. All counts were performed in triplicate.

$$Weight\;loss(\%)=\frac{Initial\;weight-final\;weight}{Initial\;weight}\times 100\%$$

#### 2.3.3. Sensory Evaluation

Use the GH/T 1408-2022 [15] fresh apple sensory evaluation method and make changes. Ten food students who had been trained in professional sensory evaluation were invited to form an evaluation team and evaluate four aspects of the grapes, color, odor, taste, and texture, with a full score of 100 points. Ten students majoring in food received 7 days of sensory evaluation training. First, the students were trained in taste with citric acid, glucose, and salt, and the students were trained in color and smell with fresh grapes and rotten grapes. Each student will taste and smell samples in a separate room. The sensory rating scale is shown in Table 1.

### 2.4. Effects of Different Concentrations of Ɛ-PL Combined with Plant Extracts on the Microbial Activity and Weight Loss of Grapes

#### 2.4.1. Effects of Different Concentrations of Ɛ-PL on the Total Number of Colonies

Same method as 2.3.1 Ten grams of grape samples treated with different concentrations of Ɛ-PL combined with plant extracts were accurately weighed and poured into a homogenizing bag, and 90 mL of sterilized saline was added. The samples were then tapped with a homogenizer for 1 min. One milliliter of homogenate was taken for 10-fold serial dilution and subsequently cultured on LB plates, and the total number of colonies was viewed in culture for 48 h. All counts were performed in triplicate.

#### 2.4.2. Effects of Different Concentrations of Ɛ-PL on the Weight Loss Rate of Grapes

Same method as 2.3.2 The concentrations of Ɛ-PL were 0.20, 0.40, 0.60, 0.80, and 1.00 g/L, and the mass concentration of the plant extract was 0.60 g/L. The weight loss rate of the grapes was calculated by dividing the initial mass minus the mass measured during storage by the initial mass.

$$Weight\;loss(\%)=\frac{Initial\;weight-final\;weight}{Initial\;weight}\times 100\%$$

### 2.5. Microbiological Analysis of Grapes

#### 2.5.1. Analysis of the Microbial Diversity of Grapes

The pulp was removed from freshly picked grapes, and the skin was placed in sterile water, ultrasonicated for 2–3 min, and filtered through a 0.22-micron filter membrane until a thin layer could be clearly seen covering the membrane, after which it was placed at −80 °C for backup. The pulp was placed directly into a −80 °C freezer until use.

The quality of the extracted genomic DNA was checked via electrophoresis on a 1% agarose gel, and the concentration and purity of the DNA were determined using a NanoDrop 2000 spectrophotometer (Thermo Fisher Scientific, Waltham, MA, USA). The above extracted DNA was used as a template for PCR amplification of the V3–V4 variable region of the 16S rRNA gene using the upstream primer 338F (5′-ACTCCTACGGGGAGGCAGCAG-3′) and the downstream primer 806R (5′-GGACTACHVGGGTWTCTAAT-3′), both of which carry barcode sequences. The samples were subjected to PCR. Three replicates were performed for each sample. Sequencing analysis was performed using Illumina’s MiSeq PE300/NovaSeq PE250 platform (Shanghai Meiji Biomedical Science and Technology Co., Shanghai, China).

#### 2.5.2. Determination of the Minimum Inhibitory Concentrations of Plant Extracts on Grapes

Determination of the minimum inhibitory concentrations of plant extracts on grapes was tested as reported by Chen et al. [16], with minor improvements. The perilla and cinnamon extracts were mixed with LB liquid media to form liquid media with concentrations of 64.00, 32.00, 16.00, 8.00, 4.00, 2.00, 1.00, and 0.50 mg/mL, and a total viable count of 1 × 10^6^ CFUs/g in bacterial sap of grapes was added to 96-well plates at a ratio of 1:100. One row of negative control (blank medium only without bacterial sap of grapes) and one row of positive control (bacterial solution of grapes without plant extracts) were placed on the same plate. Results were observed by incubation at 37 °C for 24 h. All counts were performed in triplicate.

### 2.6. Analysis of Plant Extract Constituents

#### 2.6.1. Analysis of the Flavonoid Composition

A Waters Acquity UPLC HSS T3 column (1.80 µm, 2.10 mm × 100 mm) was used with a flow rate of 0.30 mL/min, a run time of 18 min, and an injection volume of 5 µL at 30 °C. An appropriate amount of sample (approximately 0.10 g) was weighed in a 5 mL centrifuge tube, 1 mL of 80% methanol in water was added, and the tube was shaken well for 1 min. The sample was then placed in a grinder, ground, and ultrasonicated at 4 °C for 30 min. The mixture was allowed to stand at 4 °C for 60 min and then centrifuged at 8000 rpm for 10 min. The supernatant was used for analysis.

#### 2.6.2. Analysis of the Phenolic Acid Composition

Separation was conducted on an Acquity UPLC HSS C18 column (1.80 µm, 2.10 mm × 100 mm) under the following conditions: column temperature, 40 °C; flow rate, 0.30 mL/min; run time, 15 min; and injection volume, 5 µL. The appropriate amount of sample was weighed, 0.90 mL of 70% methanol in water was added, and the mixture was vortexed and centrifuged at 12,000 rpm for 10 min. The supernatant was used for analysis.

### 2.7. Comparison of the Antioxidant Properties of the Plant Extracts

#### 2.7.1. Measurement of the DPPH Free Radical-Scavenging Capacity

The DPPH free radical-scavenging capacity of the plant extracts was determined using a DPPH Free Radical-Scavenging Assay Kit (R27138 Shanghai Yuanye Biotechnology Co., Ltd., Shanghai, China). In the blank group, 0.20 mL of the nitrogen radical extract mixture, 0.90 mL of anhydrous ethanol, and 0.90 mL of DPPH were added. In the sample group, 0.20 mL of the sample mixture was added, and the remainder of the volume was the same as that in the blank group. In the control group, 0.20 mL of the sample mixture and 1.80 mL of anhydrous ethanol were added and mixed well, and then the sample mixture was incubated at room temperature without light for 30 min. The absorbance values were measured at 517 nm. The samples were assayed in triplicate. All counts were performed in triplicate.

#### 2.7.2. Analysis of the ABTS Free Radical-Scavenging Capacity

The ABTS free radical-scavenging capacity of the plant extracts was determined using an ABTS Free Radical-Scavenging Assay Kit (R32104; Shanghai Yuanye Biotechnology Co., Ltd., Shanghai, China). A total of 280 µL of ABTS working solution and 7 µL of distilled water were added to the blank group, and 280 μL of ABTS working solution and 7 μL of the sample mixture were added to the sample group. The samples were mixed and allowed to react for 2–6 min at room temperature, after which the absorbance value was measured at 405 nm. The samples were assayed in triplicate. All counts were performed in triplicate.

#### 2.7.3. Determination of the Total Antioxidant Capacity

The total antioxidant capacity of the plant extracts was determined using a total antioxidant capacity (T-AOC) assay kit (R24147-100T Shanghai Yuanye Biotechnology Co., Ltd., Shanghai, China). In the blank group, 30 µL of distilled water and 264 μL of FRAP working solution were added, and in the sample group, 30 μL of sample solution and 264 μL of FRAP working solution were added. The samples were mixed well, and the tubes were placed in a 37 °C water bath and incubated for 30 min. The absorbance value was measured at 593 nm, which was substituted into the standard curve to obtain the total antioxidant capacity. The samples were analyzed in triplicate. All counts were performed in triplicate.

### 2.8. Statistical Analysis

The results obtained from the experiments were subjected to three repeated measurements. Differences among the data were analyzed using one-way analysis of variance (ANOVA) followed by Duncan’s test (*p* < 0.05) on SPSS software (version 26.0, IBM). This post hoc test allows us to compare the differences between different treatment groups of samples at the same storage time. Before statistical data analysis, the number of colonies was expressed in logarithmic values.

## 3. Results and Discussion

### 3.1. Effects of the Type of Plant Extract on the Microbes, Weight Loss, and Sensory Quality of Grapes

#### 3.1.1. Total Number of Colonies

**Figure 1:**
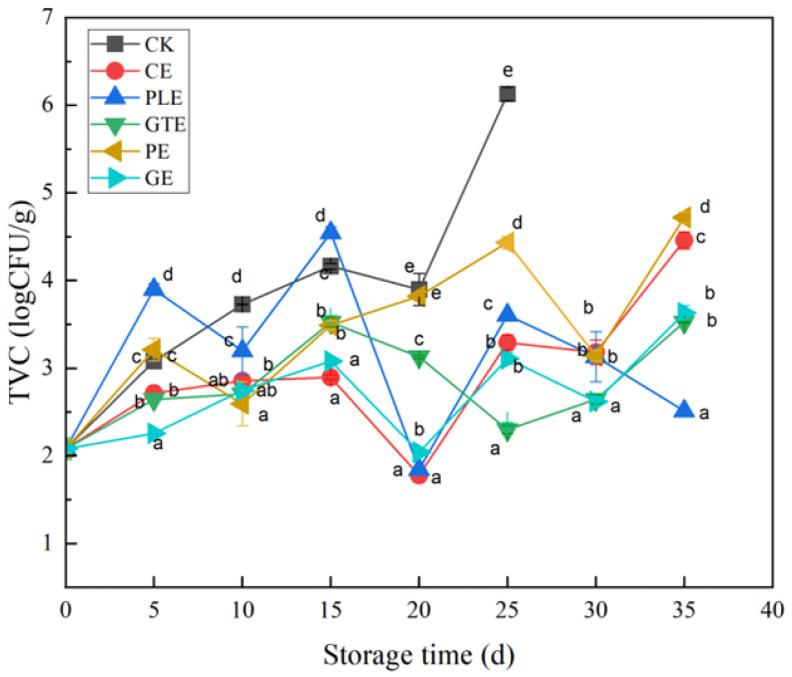
Effects of plant extract species on the total number of grapevine colonies. (CK, control; CE, cinnamon extract; PLE, perilla extract; GTE, green tea extract; PE, pomegranate peel extract; GE, ginger extract; the means with different letters (a–e) are statistically different under different treatments in the same time (*p* < 0.05)).

As shown in Figure 1, the initial total microbial count of grapes in the control group was 2.08 logCFU/g, and the total colony count reached 4.16 logCFU/g on the 15th day of storage. This value continued to increase, reaching 6.13 logCFU/g on the 25th day of storage, after which the grapes were completely spoiled. The microorganisms in the experimental groups were inhibited, and the total count increased in a fluctuating pattern, in which the inhibitory effect on microorganism growth was greater in the experimental groups treated with perilla, green tea, and ginger extracts. The total number of microorganisms in the perilla extract experimental group was 2.61 logCFU/g on the 35th day of storage, which was only 0.53 logCFU/g greater than the total number of microorganisms present at the initial time point. The total numbers of microorganisms in the experimental groups treated with green tea and ginger plant extracts were 3.52 logCFU/g and 3.63 logCFU/g, respectively, on the 35th day of storage, which were lower than the value of 4.00 logCFU/g. According to the literature, plant extracts are rich in antimicrobial compounds, such as flavonoids and phenolic acids, which have antimicrobial properties. The alcoholic extract of perilla contains perilla aldehyde, which has a good inhibitory effect on *Aspergillus niger (A. niger)*, a microbe that triggers the spoilage of grapes, with a minimum inhibitory concentration (MIC) and a minimum fungicidal concentration (MFC) of 0.25 μL/mL and 1.00 μL/mL, respectively [17]. Studies have shown that the inhibition zone diameter of cinnamon essential oil against *Bacillus subtilis(B. subtilis)* is 29.89 ± 2.99 mm, and the inhibition zone diameter against *A. niger* is 29.67 ± 0.36 mm [18]. Studies have shown that the minimum inhibitory concentration of antibacterial active ingredients in ginger extract against *B.subtilis* is 12.05 ± 2.03 mg/mL [19].

#### 3.1.2. Rate of Weight Loss

**Figure 2:**
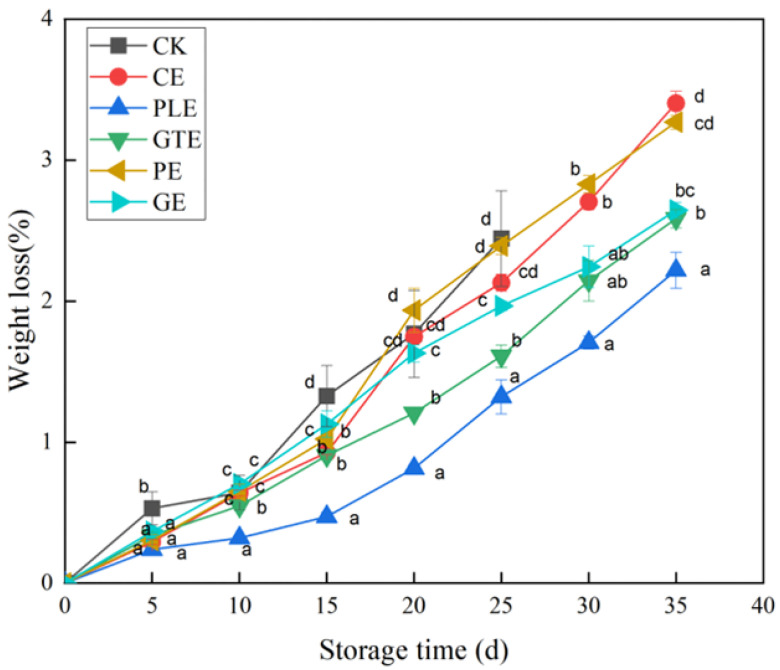
Effects of various plant extracts on the weight loss of grapes. (The means with different letters (a–e) are statistically different under different treatments at the same time (*p* < 0.05)).

As shown in Figure 2, the weight loss rates in both the control and experimental groups tended to increase linearly. The weight loss rate of the control group was greater than that of the other experimental groups on day 15 of storage, reaching 1.33%, and increased to 2.44% on day 25 of storage. The experimental groups treated with perilla and green tea extracts exhibited greater reductions in the rates of weight loss of the grapes. After 15 days of storage, the weights decreased by 0.57% and 0.65%, and the rates of weight loss increased to 1.32% and 1.43% at 25 days of storage, respectively. At 35 days of storage, the rates of weight loss remained below 2.40% in both groups. Harvested fruits still undergo various physiological and metabolic activities, resulting in the continuous loss of water and solutes and gas exchange with the environment through respiration and transpiration, leading to quality loss [5]. In the present study, changes in the grape water content and the consumption of organic matter were slowed to some extent by maceration with plant extracts. This may be because the plant extracts are rich in polysaccharides and hydroxyl groups, which can be combined with water through hydrogen bonds to form a water-holding membrane to prevent water loss from grapes. Malus ‘Donald Wyman’ crabapple extract was used to treat grapes, which could effectively reduce the weight loss rate of grapes to 15.90% and maintain the storage quality of grapes [20].

#### 3.1.3. Sensory Evaluation of Grapes After Treatment with Different Plant Extract Species

**Figure 3:**
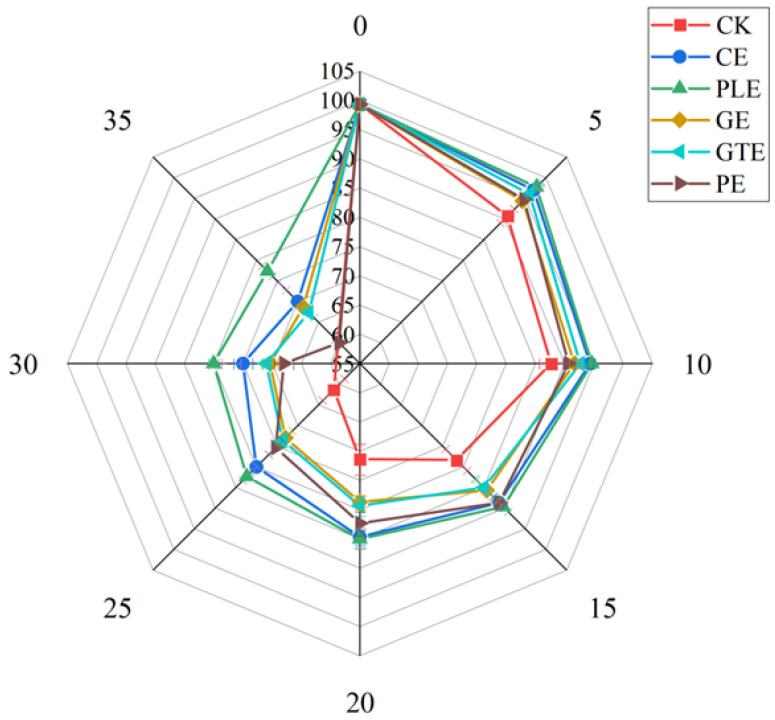
Effects of plant extract type on the sensory evaluation of grapes.

**Figure 4:**
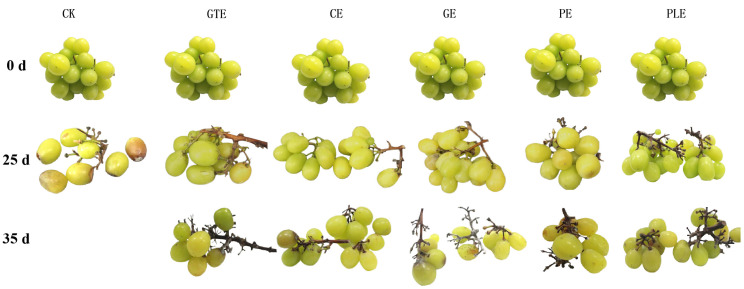
Effects of plant extract type on the appearance of grapes.

The effects of the plant extracts on the organoleptic state of the grapes are shown in Figure 3 and Figure 4. On day 0, the grapes were lime green, glossy, and plump. With prolonged storage time, the morphology, color, and other sensory characteristics of the fruits in the control group decreased significantly, and severe wilting, shedding, and rotting phenomena were observed, with the fruits ultimately completely losing their commercial value. In contrast, on the 25th day of storage, the grapes in the perilla and cinnamon experimental groups did not show any shedding or rotting phenomena and had good sensory ratings, with scores of 82 and 80, respectively. However, the experimental groups treated with ginger and pomegranate peel extracts showed partial yellowing and peeling, and the sensory scores decreased below 75. At 35 days of storage, the perilla and cinnamon experimental groups began to show partial shedding and yellowing, whereas the green tea, ginger, and pomegranate peel experimental groups presented greater than 50% shedding. These findings suggest that the perilla and cinnamon extracts had significant effects on the overall sensory quality of grapes. When an edible coating with cinnamon was applied to the surface of strawberries, the decay rate of strawberries was less than 20% at 6 days of storage, whereas the control fruit had a decay rate of more than 50% on day 3 of storage [21]. Similarly, a coating with cinnamon essential oil was used for the preservation of mango, and the experimental group did not show decay until the 10th day of storage, whereas the control group had already begun to decay by the 6th day of storage [22].

In conclusion, by investigating the effects of different plant extracts on the total number of colonies, weight loss, and sensory quality of grapes, it was found that perilla and cinnamon extracts performed better on the overall quality of grapes. Therefore, perilla and cinnamon extracts were selected for subsequent studies.

### 3.2. Effects of Plant Extracts Combined with Ɛ-PL on the Microbial Activity and Weight Loss of Grapes

#### 3.2.1. Effects of Plant Extracts Containing Different Concentrations of Ɛ-PL on the Total Number of Colonies

**Figure 5:**
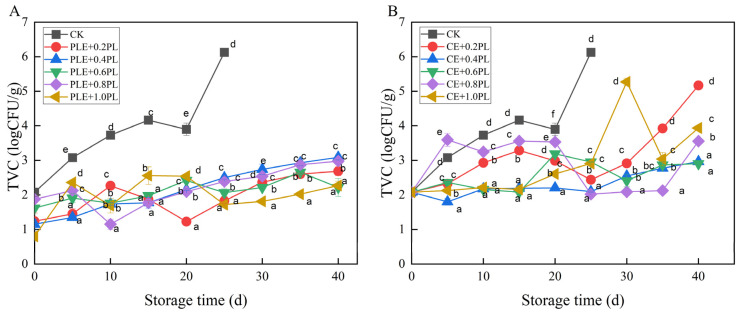
Effects of plant extracts combined with different Ɛ-PL concentrations on the number of grapevine colonies. ((**A**) perilla extract combined with different concentrations of Ɛ-PL, (**B**) cinnamon extract combined with different concentrations of Ɛ-PL; the means with different letters (a–f) are statistically different under different treatments in the same time (*p* < 0.05)).

The effects of perilla and cinnamon extracts combined with Ɛ-PL on grape preservation were further investigated. The sensory quality of the grapes in the experimental group was favorable from 0 to 40 days. On the 40th day of storage, the sensory scores of the grapes in the experimental group were higher than 80 points. As shown in Figure 5A, combinations of different concentrations of Ɛ-PL and perilla extract effectively controlled microbial growth in grapes. On the 25th day of storage, the 0.20, 0.60, and 1.00 g/L Ɛ-PL experimental groups performed better than the group treated with the perilla extract alone, with microbial counts of 1.83, 2.07, and 1.72 logCFU/g, respectively. When the storage time was further extended to 40 days, the 0.60 and 1.00 g/L Ɛ-PL experimental groups performed better, with a microbial count of 2.27 logCFU/g.

As shown in Figure 5B, cinnamon extract combined with different concentrations of Ɛ-PL also enhanced the bacteriostatic properties, but the synergistic effect on the bacteriostatic properties was less pronounced than that of the perilla extract. On the 25th day of storage, the 0.40 and 0.80 g/L Ɛ-PL experimental groups performed better, with microbial counts of 2.10 and 2.02 logCFU/g, respectively. On the 40th day of storage, the 0.40 and 0.60 g/L Ɛ-PL experimental groups performed better, with microbial counts of 2.91 logCFU/g. Figure 5 shows that the inhibitory effect on microbial growth did not increase with increasing Ɛ-PL concentration. 

#### 3.2.2. Effects of Plant Extracts Containing Different Concentrations of Ɛ-PL on the Weight Loss of Grapes

**Figure 6:**
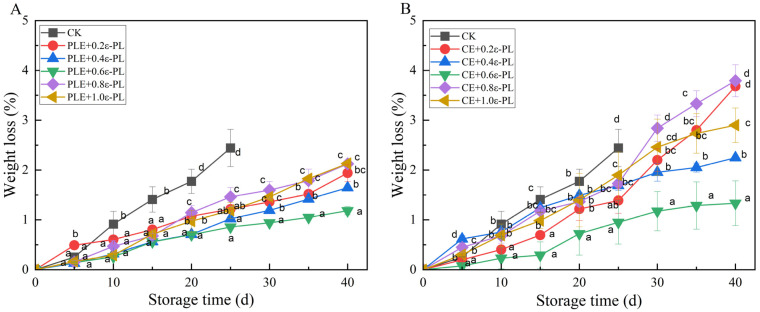
Effects of plant extracts combined with different Ɛ-PL concentrations on the weight loss of grapes. (**A**) perilla extract combined with different concentrations of Ɛ-PL, (**B**) cinnamon extract combined with different concentrations of Ɛ-PL; the means with different letters (a–e) are statistically different under different treatments at the same time (*p* < 0.05).

As shown in Figure 6A, perilla extract combined with different concentrations of Ɛ-PL effectively delayed the increase in the weight loss rate of the grapes. On the 25th day of storage, the 0.60 g/L Ɛ-PL experimental group exhibited the greatest effect, followed by the 0.40 g/L Ɛ-PL experimental group, with a weight loss rate of 0.86%, and the 0.80 g/L Ɛ-PL experimental group displayed the worst effect, with a weight loss rate of 1.46%. On the 40th day of storage, the 0.60 g/L Ɛ-PL experimental group exhibited the greatest effect, with a weight loss rate of 1.18%, and the 0.80 and 1.00 g/L Ɛ-PL experimental groups performed the worst, with a weight loss rate of 2.13%.

As shown in Figure 6B, the effect of the cinnamon extract combined with different concentrations of Ɛ-PL on the weight loss rate of grapes was weaker than that of the perilla extract. On the 25th day of storage, the 0.60 g/L Ɛ-PL experimental group performed the best, with a weight loss rate of 0.95%, and the 1.00 g/L Ɛ-PL experimental group performed the worst, with a weight loss rate of 1.90%. On the 40th day of storage, the weight loss rate of the 0.60 g/L Ɛ-PL experimental group increased to 1.33%, the weight loss rate of the 0.40 g/L Ɛ-PL experimental group was 2.25%, and all the other experimental groups had weight loss rates greater than 2.44%. In summary, the combination of 0.60 g/L Ɛ-PL with two different plant extracts produced good results. A study revealed that 0.05% Ɛ-PL was effective at slowing the reduction in the level of organic solutes in fresh-cut kiwifruit, thus slowing weight loss [23].

### 3.3. Analysis of Grape Microbial Species

#### 3.3.1. Analysis of Microbial Diversity in Grapes

**Figure 7:**
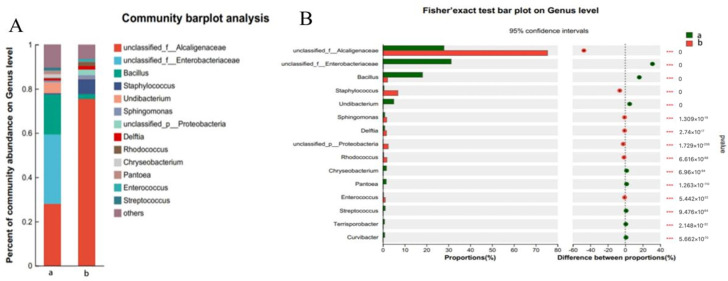
Grape microbial communities. (a denotes grape skin, b denotes grape flesh, (**A**) denotes the species community map, and (**B**) denotes the map of differences between skin and flesh microbes).

As shown in Figure 7A, the percentage of unclassified_f_Enterobacteriaceae (unclassified_f_Ent) was the highest in the pericarp, followed by unclassified_f_Alcaligenaceae (unclassified_f_Alcalig) and Bacillus. The percentage of unclassified_f_Alcalig was the highest in the pulp, at nearly 80%, followed by ylococcus (Staph). As shown in Figure 7B, the percentages of unclassified_f_Ent and Bacillus in the pericarp were significantly higher than those in the pulp, whereas the percentages of unclassified_f_Ent and Bacillus were significantly higher than those in the pulp. The percentages of unclassified_f_Alcalig and Staph were significantly lower than those in the pulp. The results revealed that the dominant genera in grape skin were unclassified_f_Ent, unclassified_f_Alcalig, and Bacillus, and the dominant genus in grape pulp was unclassified_f_Ent.

#### 3.3.2. Minimum Inhibitory Concentrations of Plant Extracts Against Grape Microorganisms

**Figure 8:**
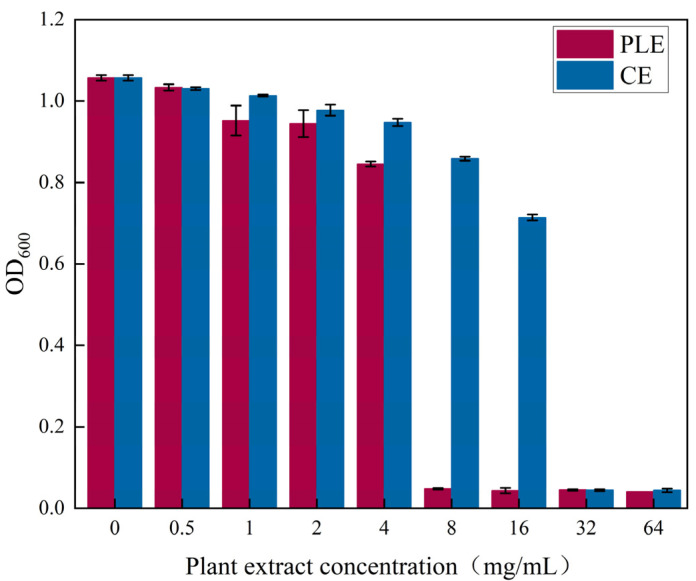
Inhibitory effects of different perilla and cinnamon extract concentrations on grape microorganisms. (OD is the absorbance value of the bacterial solution at a wavelength of 600 nm, higher values indicate that the concentration of the bacterial solution is greater).

The bacterial mixture of grapes was inoculated into LB medium and cultured to 10^6^ CFUs/g. The inhibitory effects of different concentrations of plant extracts on mixed bacteria from grapes were determined. The OD_600_ of the negative control (blank LB medium) was 0.050. As shown in Figure 8, when the concentration of perilla extract was 8.00 mg/mL, the OD_600_ of the bacterial mixture was 0.048 (OD_600_ < 0.050), and when the concentration of cinnamon extract was 32.00 mg/mL, the OD_600_ of the bacterial mixture was 0.044 (OD_600_ < 0.050). Thus, the minimum inhibitory concentrations of the perilla and cinnamon extracts on mixed grape microorganisms were 8.00 mg/mL and 32.00 mg/mL, respectively.

### 3.4. Analysis of Active Substances in Perilla and Cinnamon Extracts

The experimental data revealed that the perilla and cinnamon extracts had good effects on preserving grapes, which may be due to the presence of many flavonoids and phenolics with antimicrobial and antioxidant activities in both extracts.

#### 3.4.1. Analysis of Active Antimicrobial Substances in Perilla and Cinnamon Extracts

As shown in Table 2, the contents of flavonoid constituents such as luteolin, diosmin, apigenin, vitexin, and rutin were relatively high in the perilla extracts, with concentrations of 5.65 × 10^−3^, 3.93 × 10^−3^, 3.41 × 10^−3^, 2.47 × 10^−3^, and 1.58 × 10^−3^ μg/mg, respectively. The contents of phenolic components such as caffeic acid, protocatechuic aldehyde, gallic acid, protocatechuic acid, and coumaric acid were relatively high, with concentrations of 0.48 μg/mg, 0.33 μg/mg, 0.35 μg/mg, 0.25 μg/mg and 0.13 μg/mg, respectively. Luteolin exhibits bactericidal activity primarily by inducing oxidative stress in bacterial cells, leading to severe structural damage, inhibition of metabolic and respiratory activities, dysfunctional energy metabolism, and ultimately bacterial death [24]. The MIC values of protocatechuic acid against *Escherichia coli* (*E. coli*), *Pseudomonas aeruginosa* (*P*. *aeruginosa*), and *Staphylococcus aureus* (*S.aureus*) were 0.55, 0.30, and 0.45 mg/mL, respectively, and the MBC values were 0.60, 0.40, and 0.50 mg/mL, respectively [25]. Vitexin can regulate the hydrophobicity of the *S. aureus* surface, and the inhibitory concentration of vitexin against *S. aureus* is 0.25 mg/mL [26]. The minimum inhibitory concentration of vitexin against *P*. *aeruginosa* is 0.26 mg/mL [27]. Caffeic acid and Ɛ-PL have been used to make composite antimicrobial films with an antimicrobial film concentration of more than 3%, an inhibitory effect on *E. coli* > 91%, and an inhibitory effect on *S. aureus* > 97% [28]. The ursolic acid content in perilla extract was 1.40 × 10^−3^ μg/mg, and studies have shown that ursolic acid has some antifungal activity, with MIC values ranging from 20 to 250 mg/mL [29]. The ferulic acid content in perilla extract was 1.04 mg/kg, and the IC_50_ values of the combination of ferulic acid and coumaric acid are 215 ± 1.3 μM against *B. subtilis* and 341 ± 3.6 μM against *P*. *aeruginosa* on day 5 [30]. The content of chrysin in perilla extract was 1.20 × 10^−3^ mg/kg, and some studies have shown that the MIC values of 20 mg/mL chrysin against *Salmonella typhimurium* (*Sty*), *Vibrio cholerae* (*V. cholerae*) and *B. subtilis* are 1.25, 2.5, and 1.25 mg/mL, respectively [31].

As shown in Table 3, the contents of flavonoid constituents such as epicatechin, vanillin, morin, quercetin, and pinocembrin were relatively high in the cinnamon extract, with concentrations of 0.60, 1.63 × 10^−3^, 1.30 × 10^−3^, 1.29 × 10^−3^, and 1.01 × 10^−3^ μg/mg, respectively. The phenolic components, such as protocatechuic acid, protocatechuic aldehyde, catechin, coumaric acid, and ferulic acid, were also present at relatively high levels, with concentrations of 0.35, 0.11, 1.70 × 10^−2^, 4.48 × 10^−3^, and 3.05 × 10^−3^ μg/mg, respectively. The minimum inhibitory concentration of vanillin against *E. coli* was 2.00 mg/mL, and vanillin-treated *E. coli* cells presented damage to the membrane system, membrane depolarization, leakage of nucleic acids and proteins, and a decrease in the ATP content, the effects of which led to cell death [32]. Pinocembrin acts against *E. coli* and *S. aureus* by disrupting the integrity of the bacterial cell wall and cell membrane, inhibiting the formation of bacterial biofilms, inducing damage, and leading to increases in the activity levels of superoxide dismutase and alkaline phosphatase in bacteria [33]. The MIC and MBC of pinocembrin against *E. coli* were 1.11 and 2.22 mg/mL, whereas those of *S. aureus* were 0.56 and 1.11 mg/mL [33]. Quercetin extracted from oregano was combined with ethanolamine to synthesize a Schiff base, which significantly inhibited Gram-negative bacteria, including *P. aeruginosa* and *E. coli*, with an MIC of 0.50 mg/mL [34]. The content of kaempferol in cinnamon extract was 0.26 × 10^−3^ μg/mg, and one study used kaempferol in combination with silver nanoparticles (AgNPs), which presented MIC and MBC values of 1.25 and 2.50 mg/mL, respectively, against *S. aureus* [35]. The ellagic acid content of cinnamon extract was 2.71 × 10^−3^ mg/kg. In one study, 3,3′-di-O-methylellagic acid was extracted from African wolfberry trees, which showed the highest levels of inhibition against *E. coli*, *Sty*, and *Candida albicans* (*C. albicans*), with a concentration range of 6.25–0.20 mg/mL [36].

#### 3.4.2. Analysis of Active Antioxidant Substances in Perilla and Cinnamon Extracts

The perilla and cinnamon extracts were found to have good antioxidant effects on grapes, delaying the appearance of yellowing. A comparison of the antioxidant properties of the two plant extracts revealed that the ABTS and DPPH scavenging capacities of the perilla extract were 28.32 ± 3.13% and 52.35 ± 2.52%, respectively, and those of the cinnamon extract were 18.41 ± 0.70% and 35.62 ± 2.53%, respectively. The total antioxidant capacities of the perilla and cinnamon extracts were 1.82 ± 0.43 and 0.58 ± 0.23 mmol/L, respectively, indicating that the antioxidant capacity of the perilla extract was greater than that of the cinnamon extract. As shown in Table 2 and Table 3, many antioxidant components, such as protocatechuic acid, caffeic acid, coumaric acid, rutin, apigenin, catechin, and quercetin, are present in the perilla and cinnamon extracts. Protocatechuic acid contains two potential metal-binding sites, catechol, and carboxylic acid, showing a high affinity for metal. The antioxidant capacity of Protocatechuic acid was 10 times higher than that of α-tocopherol [37]. The two phenolic hydroxyl groups of caffeic acid have a steric hindrance effect, which promotes the transfer of electrons and hydrogen atoms, thereby giving caffeic acid excellent antioxidant capacity [38]. The contents of coumaric acid and rutin in the perilla extract were approximately 29 and 10 times greater than those in the cinnamon extract, respectively. Coumaric acid has good antioxidant activity. Coumaric acid has strong scavenging effects on hydrogen peroxide, superoxide radicals, light radicals, and peroxynitrite, and treating broccoli with coumaric acid can reduce the decline in the chlorophyll content and delay the broccoli yellowing phenomenon [39]. Rutin is a natural flavonoid glycoside with antioxidant, antibacterial, antiviral, and other pharmacological activities, of which the antioxidant effect is more significant [40]. The active component apigenin was also detected in perilla extract; apigenin is a widely distributed flavonoid, and apigenin extracted from *Teucrium polium* has a good IC_50_ for its antioxidant activity, as it scavenges DPPH free radicals at a concentration of 0.026 ± 1.52 mg/mL [41].

The literature shows that -polylysine has significant antibacterial activity. Perilla and cinnamon extracts are rich in active substances, such as coumaric acid, rutin, apigenin, catechin, and quercetin, indicating that they have good antioxidant properties. In addition, the extracts of perilla and cinnamon also contain protocatechuic acid, vitexin, and other substances, which can play an auxiliary antibacterial role. The synergistic effect of antibacterials and antioxidants guarantees the quality of grapes.

## 4. Conclusions

In this study, the effects of five different plant extracts on the microbial activity, weight loss, and sensory quality of grapes were investigated. The perilla and cinnamon extracts effectively inhibited the growth of microorganisms, decreased weight loss, and delayed yellowing and abscission. The effects of the perilla and cinnamon extracts combined with different concentrations of Ɛ-PL on microorganisms and the weight loss of grapes were further investigated, and the results revealed that 0.6 g/L Ɛ-PL combined with perilla and cinnamon extracts had better effects on controlling the microorganisms and weight loss of grapes than the other extracts. The microbial colony numbers decreased to 2.27 and 2.91 logCFU/g, and the weight loss rates decreased to 1.18% and 1.33%, respectively, after 40 d of storage. The minimum inhibitory concentrations of the perilla and cinnamon extracts against grape microorganisms were 8.00 and 32.00 mg/mL, respectively. An analysis of the active components of the two plant extracts revealed that caffeic acid, protocatechuic aldehyde, gallic acid, protocatechuic acid, and coumaric acid were more abundant in the perilla extract, and epigallocatechin, protocatechuic acid, protocatechuic aldehyde, catechin, and coumaric acid were more abundant in the cinnamon extract, revealing the mechanisms of action of the perilla and cinnamon extracts in the preservation of grapes. The combination of plant extracts and Ɛ-PL provides a theoretical basis and practical guidance for grape preservation. In the future, plant extracts and Ɛ-PL will be further extended to other fruit preservation applications.

## Figures and Tables

**Table 1:** The sensory scores of colors, odors, and textures of grapes stored for 0, 5, 10, 15, 20, 25, 30, and 35 days were evaluated by 10 students.

Metric	Characteristics	Score
Color	Yellow-green, shiny skin, bright color	14–25
	Pale white, skin gloss darkened	7–13
	Yellow-brown skin, matte	1–6
Odor	Strong rose scent and no other off-flavors	14–25
	Rose fragrance fades	7–13
	Sour and moldy, with an ethylene flavor	1–6
Taste	Sweet and tasty, delicate taste	14–25
	Sweetness fades	7–13
	Sour, not edible	1–6
Texture	The fruit is full and without shrinkage, and the peel and pulp have good adhesion	14–25
	Fruit is slightly softer, slightly wrinkled, and the adhesion between the peel and the pulp becomes worse	7–13
	Fruit is obviously soft and wrinkled, with moldy spots, and the adhesion between peel and pulp is poor	1–6

**Table 2:** Active constituents of *Perilla frutescens* extract.

Serial Number	Component	Content (μg/mg)	Serial Number	Component	Content (μg/mg)
A1	Luteolin	5.65 × 10^−3^	B1	Caffeic acid	0.48
A2	Diosmin	3.93 × 10^−3^	B2	Protocatechuic Aldehyde	0.33
A3	Apigenin	3.41 × 10^−3^	B3	Gallic acid	0.35
A4	Vitexin	2.47 × 10^−3^	B4	Protocatechuic acid	0.26
A5	Rutin	1.58 × 10^−3^	B5	Coumaric acid	0.13
A6	Chrysin	1.20 × 10^−3^	B6	Syringic acid	1.18 × 10^−2^
A7	Vanillin	0.66 × 10^−3^	B7	Salicylic acid	6.41 × 10^−3^
A8	Genistin	0.58 × 10^−3^	B8	P-coumaric acid	3.34 × 10^−3^
A9	Baicalin	0.51 × 10^−3^	B9	Quinic acid	1.91 × 10^−3^
A10	Quercetin	0.17 × 10^−3^	B10	Ursolic acid	1.40 × 10^−3^
A11	Mulberry Pigment	0.16 × 10^−3^	B11	Ferulic acid	1.04 × 10^−3^
A12	Astragalin	0.15 × 10^−3^	B12	Catechin	1.05 × 10^−3^
A13	Daidzin	0.11 × 10^−3^	B13	Sinapic acid	0.05 × 10^−3^

**Table 3:** Active components of cinnamon plant extracts.

Serial Number	Component	Content (μg/mg)	Serial Number	Component	Content (μg/mg)
A1	Epicatechin	0.60	B1	Protocatechuic acid	0.35
A2	Vanillin	1.63 × 10^−3^	B2	Protocatechuic Aldehyde	0.11
A3	Morin	1.30 × 10^−3^	B3	Catechin	1.70 × 10^−2^
A4	Quercetin	1.29 × 10^−3^	B4	Coumaric acid	4.48 × 10^−3^
A5	Pinocembrin	1.01 × 10^−3^	B5	Ferulic acid	3.05 × 10^−3^
A6	Hyperion	0.78 × 10^−3^	B6	Ellagic acid	2.71 × 10^−3^
A7	Icariin	0.52 × 10^−3^	B7	P-coumaric acid	2.59 × 10^−3^
A8	Quercetin 3-O-a-L-rhamnopyranoside	0.39 × 10^−3^	B8	Syringic acid	2.24 × 10^−3^
A9	Vitexin	0.38 × 10^−3^	B9	Salicylic acid	1.83 × 10^−3^
A10	Diosmin	0.28 × 10^−3^	B10	Sinapic acid	0.58 × 10^−3^
A11	Kaempferol	0.26 × 10^−3^	B11	Quinic acid	0.18 × 10^−3^
A12	Rutin	0.16 × 10^−3^	B12	Ursolic acid	0.15 × 10^−3^
A13	Hesperidin	0.10 × 10^−3^	B13	Caffeic acid	0.10 × 10^−3^

## Data Availability

The original contributions presented in this study are included in the article. Further inquiries can be directed to the corresponding author.
